# Beyond the smiley face: applications of structural DNA nanotechnology

**DOI:** 10.1080/20022727.2018.1430976

**Published:** 2018-01-25

**Authors:** Aakriti Alisha Arora, Chamaree de Silva

**Affiliations:** a School of Medicine, Mercer University, Macon, GA, USA; b Department of Physics, Mercer University, Macon, GA, USA

**Keywords:** DNA nanotechnology, DNA origami

## Abstract

Since the development of DNA origami by Paul Rothemund in 2006, the field of structural DNA nanotechnology has undergone tremendous growth. Through DNA origami and related approaches, self-assembly of specified DNA sequences allows for the ‘bottom-up’ construction of diverse nanostructures. By utilizing different sets of small ‘staple’ DNA strands to direct the folding of a long scaffold strand in diverse ways, DNA origami has particularly been incorporated into a variety of prototypical applications beyond the two-dimensional (2D) smiley face. In this review, the basis of DNA nanotechnology, methods of self-assembly, and Rothemund’s DNA origami breakthrough are discussed first. Next, some of the most promising applications of structural DNA nanotechnology since 2006 are summarized. These include utilizing DNA origami as a tool for creating 3D nanostructures (including DNA bricks), as well as structural (ligand capsid binding, viral capsid binding, DNA NanoOctahedron, DNA mold, photonic devices, energy transfer units), and dynamic (DNA box-with-lid, DNA nano-robot, DNA barges, amphipathic DNA structures, DNA nanocircuits) applications of DNA origami.

## 1. Introduction

**Figure 1.**
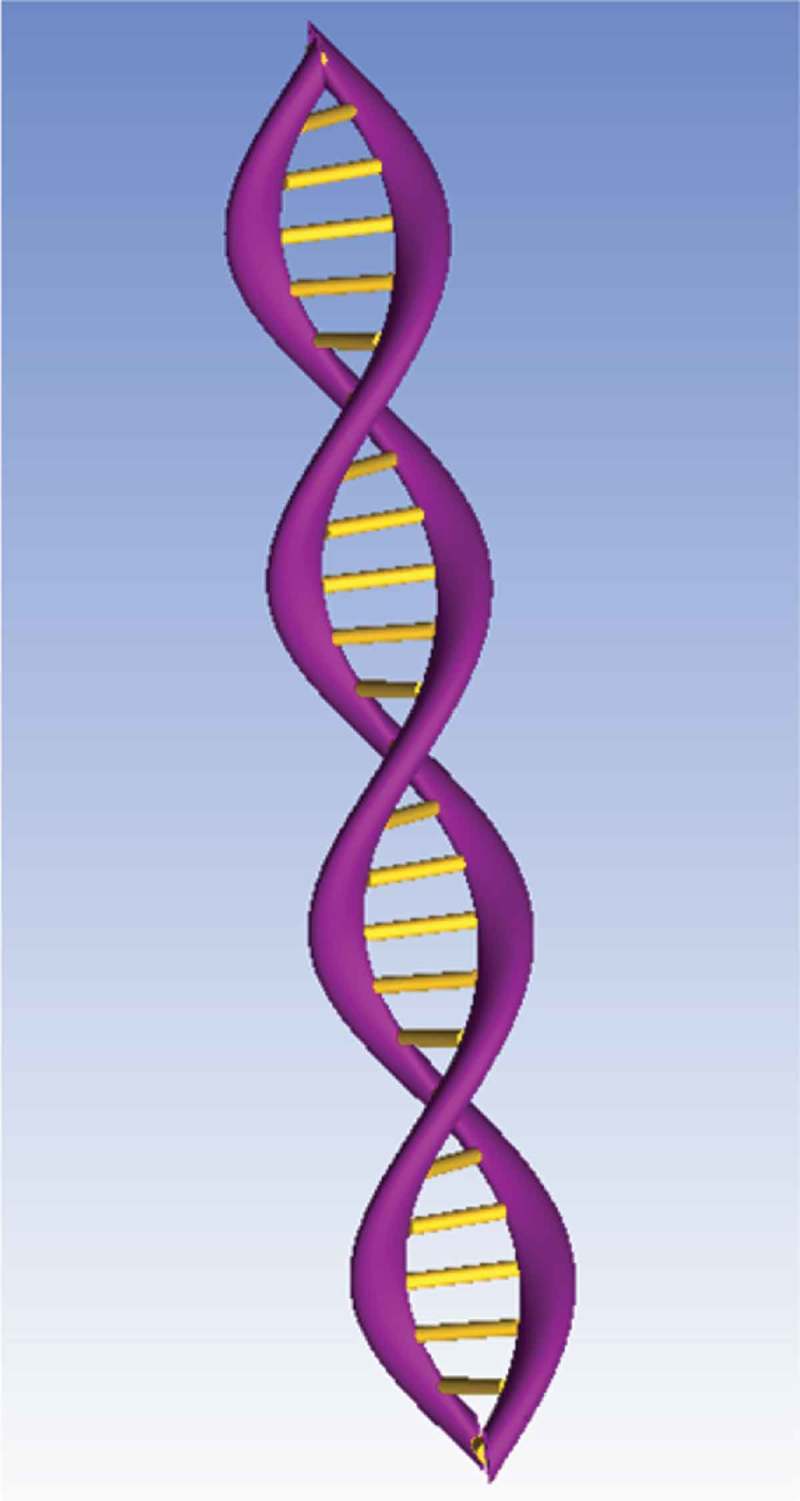
3D DNA double-helix structure comprised phosphate backbones (purple) and hydrogen bonds between nitrogenous bases (yellow). Created using ANSYS software.

A multitude of new DNA applications have been introduced since the discovery of the double helix structure in the mid-twentieth century (Figure 1). One application that is proving to be promising in a variety of fields is DNA nanotechnology. DNA nanotechnology refers to the design, study, and application of synthetically created DNA nanostructures. The physical and chemical properties of DNA, rather than its genetic properties, are particularly malleable for various applications in the field of DNA nanotechnology [1]. In other words, the field of DNA nanotechnology aims to design synthetic DNA constructs that exhibit structures and functions that are not found for DNA in nature. Ultimately, natural properties of DNA are harnessed as tools that can be manipulated and applied in a variety of settings related to the field of biotechnology.

The property of self-assembly, as per Watson-Crick base pairing, has largely been exploited in the field of DNA nanotechnology. The process of self-assembly is representative of what naturally occurs in DNA. That is, two single strands of known DNA sequences will self-assemble if they have complementary nitrogenous base sequences. Self-assembly is deemed ‘bottom-up’ approach which exploits local interactions between components within a system to yield a desired structure. A ‘bottom-up’ approach allows for a more precise, controlled, and predictable DNA nanostructure [2,3]. This is opposed to a ‘top-down’ approach, which uses external intervention to create an object by removing or adding material in a spatially controlled manner [3]. One advantage of self-assembly is the potential for molecular precision, which is difficult to achieve with ‘top-down’ approaches such as lithography. ‘Bottom-up’ approaches ultimately allow for the faster manufacturing of smaller nanostructures [2]. However, disadvantages of the self-assembly exist; first, the technique often leads to less than 100% yield of the desired product due to mis-assembly or mis-folding. Second, everything about the structure must be specified through local interactions, which requires many unique building blocks so that sufficient information becomes available; this can often be very expensive.

The self-assembly process of DNA was first showcased with the creation of 2D nucleic acid junctions and lattice shapes [4]. These junctions were developed as clusters; the clusters were linked directly to each other, or with interspersed linear DNA pieces (later coined as ‘sticky ends’) [4]. Subsequently, creation of immobile branched junctions allowed for a building framework upon to which other molecules could be attached [5]. Specifically, a closed cube-like structure containing six faces, eight vertices, and 12 double helical edges was developed [5]. Ultimately, 2D crystalline DNA forms were developed from synthetic DNA double crossover molecules [6]. ‘Sticky ends’ of DNA allowed for intermolecular interactions between each unit, leading to the formation of specific patterned DNA crystals [6].

## 2. 3D DNA origami

The DNA origami method is a particularly successful example of exploiting the self-assembly property of DNA to create assemble diverse 2D and 3D nanostructures. Introduced by Paul Rothemund in 2006, the origami method design utilizes short stranded DNA ‘staple’ strands that direct the folding of a long single-stranded DNA ‘scaffold’ strand into desired shapes and patterns. These shapes and patterns include smiley faces, squares, five-pointed stars, and disks [3]. The multitude of shapes and patterns were constructed in five steps. First, the desired shape was showcased as a geometric model of DNA. The shape was then filled by an even number of parallel double helices. Next, a single long-scaffold strand was folded in a back-and-forth pattern, so that it became part of each pair in the helices. Third, a set of staple strands that were complementary to the scaffold strand were added, and created periodic crossovers that folded the scaffold in desired areas based on the shape. The twists of these scaffold crossovers were then calculated, and positions were changed to relieve strain. Finally, pairs of adjacent staples were merged to yield fewer and longer staple strands. The DNA origami maintained proper stoichiometric concentrations of DNA strands, which allowed for the formation of 2D structures in a much higher yield than was possible before [3]. The DNA origami technique provided the foundation for 2D applications of DNA origami, which further provided the foundation for constructing 3D structures.

**Figure 2.**
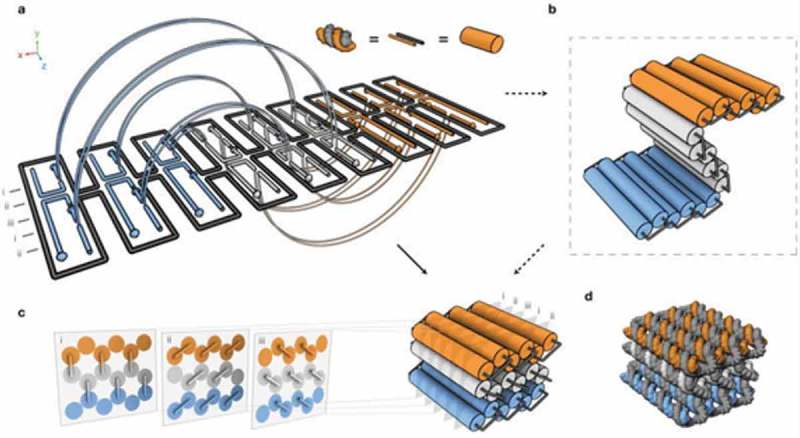
Design of the three-dimensional DNA origami: (a) Scaffold strands are depicted in grey, and staple strands are depicted in orange, white, and blue. Double helices run parallel to the Z-axis to form unrolled 2D target schema. Staple crossovers bridge layers together (semi-circular arcs), (b) cylindrical model of the intermediate, (c) 3D cylinder model of folded target shape; honeycomb arrangement is shown in cross sections (with i-iii indications). These helices are parallel to the x/y plane, (d) Atomistic model of (c). Reproduced with permission.

**Figure 3.**
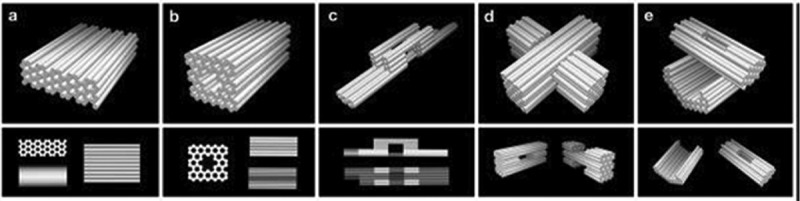
Three-dimensional DNA origami shapes: (a) monolith, (b) square nut, (c) railed bridge, (d) slotted cross, (e) stacked cross. Reproduced with permission.

Novel 3D structures were composed by forming pleated layers of helices and immobilizing them to a honeycomb lattice [7]. Double helices were designed using scaffold and several different staple strands. These helices can be initially envisioned as a single two-dimensional layer that is folded into an appropriate three-dimensional approximation of the desired shape by engineering staple crossovers to bridge different layers together (thus producing a pleat). The 2D representation created is simply a way of conceptualizing how a single scaffold strand can fold into a 3D shape; it itself may never exist as a 2D sheet during the folding process. Adjacent helices are connected by loops of unpaired scaffold strand. By arranging parallel helices as different layers, a honeycomb lattice structure results (Figure 2) [7]. Examples of structures designed include the square nut, railed bridge, genie bottle, stacked cross, slotted cross, and monolith shapes (Figure 3) [7]. Multi-layer, highly compact lattice patterns have also been developed based on the square lattice [8]. Additionally, multi-layer lattice patterns curved into barrels of controlled diameter have been developed, with the potential to create electronic or optic wires at the molecular level [9].

Other DNA structures utilizing such novel complex curvature have similarly been designed [10]. A 3D space to outline curved surfaces was used to manipulate DNA such that it bent around the contours of any particular target object. For instance, many 2D concentric rings of DNA were designed and combined to outline a desired shape. Periodic crossovers between the helices were constructed, such that when the DNA helices bent an ‘in-plane’ curvature was achieved. A scaffold crossover that wound with a single strand of every helix was formed afterward. Every time the scaffold moved from one ring to the next, it served as a scaffold crossover. Staple strands were designed to produce more crossovers and stabilize the structure. In order to achieve an ‘out-of-plane’ curvature, the position of crossover points was shifted between the double helices. The shift allowed for an off-set in a particular helix from the rest, and the natural B-form twist was lost. Now, the latitudinal curvature (2D concentric rings or ‘in-plane’ curvature) and longitudinal curvature (‘out-of-plane’ curvature) was created. Three dimensional structures such as nanoflasks, spherical shells, and ellipsoidal shells have been generated using this technique [10].

**Figure 4.**
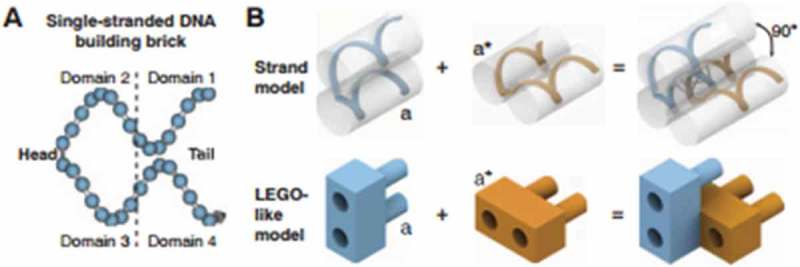
A DNA brick comprised four 8-nt domains; two of these domains are ‘head’ domains while the other two are ‘tail’ domains. Reproduced with permission.

Last, DNA bricks have been designed using 32-nucleotide (nt) synthetic DNA strands to create 3D structures [11]. A DNA brick comprised four 8-nt domains; two of these domains are ‘head’ domains while the other two are ‘tail’ domains (Figure 4). The head domains bound to the complementary tail domains, creating a two-brick assembly with a 90° dihedral angle. Utilization of shorter DNA brick strands allowed for the assembly of a multitude of complex structures, without the need of a scaffold strand. In order to demonstrate the self-assembly of a DNA cuboid structure, a new ‘voxel’ unit was implemented. In the context of DNA bricks, a voxel is defined as a structure with dimensions of 2.5 × 2.5 × 2.7 nm, and a molecular canvas as a structure with dimensions of 10 × 10 × 10 voxels. These units were identified in order to select different subsets of bricks from the molecular canvas to ultimately produce 102 novel shapes. Overall, the bricks provided a simple motif that allowed for a modular design, where each brick could be removed or added independently. Furthermore, the process was robust in sequence composition (as random sequences were used), strand synthesis (as the protocol did not require purified DNA), and stoichiometry (unlike previous efforts, no strict control was necessary). These assets will allow for new biotechnology such as programmable molecular probes, smart drug delivery particles, and photonics applications in the future [11].

## 3. Applications of DNA origami

### 3.1. Structural applications

Since Rothemund’s discovery, many studies have expanded on DNA origami techniques. For instance, origami models have been constructed in order to further understand cellular interactions. A rectangular shaped DNA tile was designed using the DNA origami method in order to understand distance-dependent multivalent ligand binding effects [12]. These multivalent binding interactions refer to the immobilization of ligands onto a DNA tile, which then bind to receptors on a specific target molecule. Immobilization was achieved through ‘closed loops’ on the DNA helices, and aptamer sequences (oligonucleotides that bind to the ligand) were incorporated onto the closed loops to allow for binding of the ligand onto the DNA tile. Multiple-affinity ligands were then integrated onto a DNA origami rectangle with precise nanometer spatial control. This allowed controlled distance between each ligand on the tile. Next, a 5-helix-bundle DNA structure was constructed in which two ligands protruded off of the tile and were separated by 2 nm, 3.5 nm, 5.3 nm, or 6.9 nm distances. Hence, by controlling the spatial components using DNA origami, the biomolecular interactions involved were also able to be controlled for [12]. Ligand binding interactions between DNA and other macromolecules such as proteins can additionally be understood using DNA origami methods.

More recently, larger structural gigadalton-scale structures have been developed by natural assembly methods along with DNA origami methods [13]. Initially, the DNA sequence encodes the shape of DNA building blocks and the interaction motifs between the building blocks control higher-order assembly [13]. Planar rings measuring 350 nm in diameter and with a mass of 330 megadaltons, thick tubular, bacillus-like structures, and 3D polyhedral structures measuring 450 nm with a mass of 1.2 gigadaltons were developed with yields up to 90% [13]. Similarly, using a ‘fractal assembly method’, sophisticated DNA origami arrays nearing 0.5 um with ~8,704 pixels have been developed [14].

Understanding ligand binding specifically between a virus capsid protein and a DNA origami tile has recently shown promise in the field of drug delivery [15]. A viral capsid was precisely immobilized through complementary sequence attachment to the ‘probe’ single-stranded sequence extending from staple strands on the DNA tile. The product yield was high while the binding pattern was predictable, both assets for conducting future biotechnology research with these structures. Ultimately, the ability of the tile to reliably recruit a massive molecular cargo was established [15]. These results are promising, and can be used in the construction ordered biomolecular systems that incorporate large biomolecular complexes such as viruses and ribosomes. For instance, drugs, catalysts, and other molecular cargo could be attached to an immobilized capsid protein, and subsequently get delivered into the cell [15]. Exploiting this ability may show immense promise in the field of biomedicine, especially regarding targeted drug delivery application.

Furthermore, recent development of mass-produced scaffold molecules via bacteriophages have shown promise in developing cost-effective DNA origami structures of any length [16]. Within a shaker-flask culture, bacteriophages produce a ‘precursor’ DNA which contains target strand sequences and self-excising Zn^2+^ dependent ‘cassettes’ containing DNA-cleaving DNA enzymes, to produce single strands that can be modifiable within any existing DNA origami design framework [16].

Inspired by a virus, and a specific application designed for biomedical application use, the NanoOctahedron structure was developed wherein a DNA nanostructure is enveloped in a lipid bilayer [17] . The nanostructure was constructed using traditional 3D DNA origami techniques, involving self-assembly of scaffold DNA and staple strands. In this case, single-stranded handles were designed to attach lipid-conjugated oligonucleotides, allowing the lipid bilayer to surround the NanoOctahedron. The lipid-bilayer exhibited less degradation and immune response activation *in vitro* and *in vivo* as compared to non-enveloped NanoOctahedrons. Therefore, this nanostructure may prove useful for biomedical applications such as tumor detection [17].

While the DNA barge involves completely organic structures, one novel 3D DNA origami application involving inorganic nanostructures is the DNA mold [18]. A DNA mold is a structure that enables the synthesis of inorganic nanostructures that have a prescribed 3D shape. When anchored in a DNA mold, an Au ‘seed’ grew into a larger metal and fills the entire cavity [18]. Ultimately, the seed grew into the prescribed 3D shape of the DNA mold. The DNA mold comprised an open-ending nanostructure barrel, with two lids attached to the barrel after insertion of the Au seed. The Au seed binds to the barrel via an anti-handle, which is complementary to DNA handles within the interior barrel. In some cases, under specific chemical conditions, the Au seed is able to grow into an Ag nanoparticle. These shapes were created at a resolution of sub-5 nm, allowing for a variety of applications in photonics [18].

The potential of DNA nanostructures in the fields of energy transfer and photonics is beginning to be uncovered [19]. For example, some nanostructures are able to immobilize components found within energy transfer systems while facilitating electron flow, much like the photosynthetic machinery of plants and other organisms is able to accomplish [19,20]. Creating DNA pegboards enabled positioning of light-harvesting complexes in close proximity with charge transfer units [19]. DNA molecular pegboards are structures that allows for the precise placement of molecules at predetermined locations [21]. This ability was illustrated by designing a DNA photonic device capable of light harvesting and energy transfer [22]. The device was developed by arranging charge separation units and antenna molecules (which are necessary for long-range energy transfer) onto a DNA template [22]. The positioning of dyes was controlled to create multichromophoric assembles, which allowed for the visualization of multi-step energy transfer. The staple strands of the DNA tile were labeled with fluorophores. When the origami structure was structured in a certain way, energy transfer was facilitated. In addition to the applications of energy transfer, dynamic applications of DNA nanotechnology have recently emerged.

### 3.2. Dynamic applications

As an example of a dynamic (moving) machine constructed from DNA origami, a DNA box with dimensions 42 × 36 × 36 nm^3^ has been developed [23]. The box was designed to be opened in the presence of external DNA ‘keys’ or oligonucleotides. Six different DNA origami sheets comprised the cuboid box structure, while staple strands connected the sheet edges together. The DNA lid was itself one of the origami sheets, and contained DNA duplexes with sticky-end ‘toe-hold’ extensions. This enabled the binding of the ‘key’ oligonucleotides. Once the key was present, the opening and closing of the DNA lid on the box was monitored using fluorophores. The act of opening and closing suggests the potential of uncovering nanocargo delivery methods [23]. Similarly, a DNA nanoscale vault has been recently developed. Enzymes are loaded within the vault, and a multi-lock mechanism controls the opening and closing of the vault for potential substrate-enzyme interactions [24].

Another 3D DNA structure that can exhibit controlled opening and closing is the DNA nanorobot. A DNA nanorobot structure was created in the shape of a hexagonal barrel with dimensions 35 nm × 35 nm × 45 nm [25]. The barrel had two covalently attached domains in the rear, which were attached using single-stranded scaffold hinges. At the front of the structure, staples containing DNA aptamers fastened the barrel. In order to allow quick response to specific proteins, a lock mechanism that would only open in response to antigen ‘keys’ was designed. A myriad of different molecular payloads could consequently be loaded into the nanorobots, although the release of these payloads would be controlled by an aptamer-encoded gate. Moreover, the nanorobots were loaded with antibody fragments, which increased cell-signaling stimulation in tissue culture. Because of the specificity and controlled opening of the nanorobot, this 3D structure demonstrates to be a viable tool for initiating major changes in the cell [25].

As nanorobots possess the ability for controlled dynamic movement, molecular spiders allow for more complex behavior [26]. Molecular spiders consist of a streptavidin body with three catalytic legs comprised deoxyribozymes [26]. The environment onto which the spider walks is a precisely defined DNA origami landscape. The landscape and complementary oligonucleotides allow for the spider to autonomously ‘start’, ‘follow’, ‘turn’, and ‘stop’ [26]. The track comprised DNA substrates, which are bound to the deoxyribozyme legs; the deoxyribozyme cleaves the substrate, and creates two short products [26]. Cleavage allows for the dissociation of the legs, which could then associate with the next substrate, allowing the spider to move. The spider begins to ‘walk’ after introduction with an ssDNA trigger, but once it reached a stop site on the landscape, it stops moving. The spider was tactfully designed to exhibit ‘memory’; that is, the deoxyribozyme leg dissociates faster if it reaches a site upon which it had previously visited. Hence, molecular spiders have the potential to provide the basis for initiating programmable behaviors of nanostructures in various complex environments [26].

The DNA barge showcases the potential of DNA origami to dynamically interact with a more specific complex environment; that is, the biological membrane environment. Cholesterol-labeled DNA origami barges were created to interact with the phospholipid bilayer in a manner similar to that of membrane proteins [27]. A rectangular DNA origami tile based on Rothemund’s design was created in which single-stranded DNA overhangs extended from staple strands on one side [3]. These overhangs were complementary to cholesterol-labeled anchor strands, which produced a hydrophobic surface through which the barge was able to interact with the phospholipid bilayer. The overhangs also recruited a fluorescently labeled cargo strand for purposes of tracking; the cargo could be reversibly removed and re-loaded by adding appropriate DNA oligonucleotides. This toe-hold extension was utilized for tracking purposes. To demonstrate the barges’ ability to bind and unbind to the bilayer, single-particular tracking was used. Specifically, microscopy showed that the barges bound to the lipid bilayer relatively quickly, and remained bound to the bilayer while undergoing 2D Brownian motion. These barges could thus be used as probes, illustrating membrane regions with high accuracy. In addition, these barges have potential for drug delivery, as seen by their ability to detach from the membrane, oligomerize with other DNA barges, and load and unload nanocargo [27].

Furthermore, other amphipathic DNA origami structures have been created. Transmembrane channel structures in lipid bilayers were created via a transmembrane ‘stem’ that pierced the length of the lipid membrane was created along with a barrel-shaped ‘cap’ [28]. The ‘cap’ was attached to the membrane through 26 cholesterol moieties [28]. The physiological functionality of natural ion channels was mimicked, such that conductances of one nanosiemens and channel gating were achieved [28]. Similarly, a transmembrane molecular valve which controls gated transport of material across a phospholipid bilayer has been created [27]. The valve is created from seven linked DNA strands, and binds to a ligand [29]. Once bound, the valve opens up the channel, selectively allowing positively and negatively charged small organic molecules to pass. Another amphipathic DNA origami structure consisting of cholesterol anchors on top of a flat membrane binding interface, containing ordered arrays on the membrane due to oligonucleotide overhangs present laterally [29]. Ultimately, this structure is capable of deforming free-standing lipid membranes while showing similar biological activity to coat-forming proteins [30]. Last, amphipathic DNA origami rods with six DNA helices were created along with hydrophobic cholesteryl-ethylene glycol anchors and fluorescently labeled. The nanorods coated membranes of different phospholipid compositions and exhibited translational and rotational diffusion between different domains as visualized by the fluorescent labeling [31]. Ultimately, as more studies are conducted, these examples of amphipathic DNA origami structures show promise as physiologically relevant biostructures.

Interestingly, the metallization of small DNA origami templates has shown promise for the use of circuit fabrication in DNA related nanocircuits [32]. Using an electroless deposition process, kg was seeded onto the templates and then the templates were plated with gold until ultimately unremitting deposition of metal occurred while maintain the original branched DNA origami structure [32]. Similarly, gold nanorod seeds and anisotropic plating was employed to metallize DNA templates with improved control of the final structure [33]. Gold nanorods were used to create a seed layer, and electroless gold deposition as used in between the seeds to create narrow (13–29 nm) and conducive (resistivity values reached as low as 8.9 × 10–7 Ω·m) nanowire [33].

In addition to its use in nanocircuitry, DNA origami has also been shown to serve as a natural interface within living systems; that is, as a biocomputing method [34]. DNA origami based nanoscale robots were created, and their interactions in living cockroaches were utilized to switch molecular payloads (‘logic gates’) either ‘on’ or ‘off’ [34].

## 4. A field with great promise

Since the inception of the DNA nanotechnology field, DNA is now able to be manipulated for its structural prowess and technological purposes in addition to its inherent genetic capabilities. In many respects, DNA origami encompasses a combination of creativity along with scientific principles. Utilizing this creativity allows for promising results in molecular biology, chemistry, physics, and virtually any scientific field. Synthetically derived DNA nanostructures have the potential to lead to major scientific breakthroughs. For instance, not only has DNA origami shown to be an asset for intracellular process such as ligand binding, eventual nanocargo delivery (aiding in drug-delivery biomedical research), but it also facilitates novel energy transfers (aiding in energy renewal research). Novel applications in DNA origami have therefore undoubtedly changed the field of biotechnology and the future of biomedicine. However, it is essential to reduce cost of materials such that applications such as drug delivery become more cost-effective and realistic. Additionally, future studies are working toward ensure stability of DNA nanostructures in living cells and tissues, where physiologically low ionic strength and nucleases can cause unfolding and degradation of structures. Last, studies in this field are moving toward designing more dynamic machines and exploit mechanical properties of DNA nanostructures to do relevant and useful work. This field is still very young, and there is still untapped biomedical potential to discover. However, much of the current research points toward a bright future for the use of DNA origami in mainstream science.
